# Hypoxic enhancement of exosome release by breast cancer cells

**DOI:** 10.1186/1471-2407-12-421

**Published:** 2012-09-24

**Authors:** Hamish W King, Michael Z Michael, Jonathan M Gleadle

**Affiliations:** 1 Renal Department, Flinders Medical Centre, Flinders University School of Medicine, Bedford Park, South Australia, 5042, Australia; 2 Department of Gastroenterology and Hepatology, Flinders Medical Centre, Flinders University School of Medicine, Bedford Park, South Australia, 5042, Australia

**Keywords:** Hypoxia, Exosomes, Breast cancer cells, Nanoparticle tracking analysis, Nanosight, Exoquick^TM^

## Abstract

**Background:**

Exosomes are nanovesicles secreted by tumour cells which have roles in paracrine signalling during tumour progression, including tumour-stromal interactions, activation of proliferative pathways and bestowing immunosuppression. Hypoxia is an important feature of solid tumours which promotes tumour progression, angiogenesis and metastasis, potentially through exosome-mediated signalling.

**Methods:**

Breast cancer cell lines were cultured under either moderate (1% O_2_) or severe (0.1% O_2_) hypoxia. Exosomes were isolated from conditioned media and quantitated by nanoparticle tracking analysis (NTA) and immunoblotting for the exosomal protein CD63 in order to assess the impact of hypoxia on exosome release. Hypoxic exosome fractions were assayed for miR-210 by real-time reverse transcription polymerase chain reaction and normalised to exogenous and endogenous control genes. Statistical significance was determined using the Student T test with a *P* value of < 0.05 considered significant.

**Results:**

Exposure of three different breast cancer cell lines to moderate (1% O_2_) and severe (0.1% O_2_) hypoxia resulted in significant increases in the number of exosomes present in the conditioned media as determined by NTA and CD63 immunoblotting. Activation of hypoxic signalling by dimethyloxalylglycine, a hypoxia-inducible factor (HIF) hydroxylase inhibitor, resulted in significant increase in exosome release. Transfection of cells with HIF-1α siRNA prior to hypoxic exposure prevented the enhancement of exosome release by hypoxia. The hypoxically regulated miR-210 was identified to be present at elevated levels in hypoxic exosome fractions.

**Conclusions:**

These data provide evidence that hypoxia promotes the release of exosomes by breast cancer cells, and that this hypoxic response may be mediated by HIF-1α. Given an emerging role for tumour cell-derived exosomes in tumour progression, this has significant implications for understanding the hypoxic tumour phenotype, whereby hypoxic cancer cells may release more exosomes into their microenvironment to promote their own survival and invasion.

## Background

Exosomes are biological nanovesicles (30 – 100 nm in diameter) constitutively released by cells through the fusion of multivesicular endosomes with the plasma membrane and subsequent release of intraluminal vesicles into the extracellular environment [1,2]. Exosomes contain a wide range of functional proteins, mRNAs and microRNAs (miRNAs) [3-5], providing a novel paracrine signalling mechanism during important physiological processes, including tumour progression. Exosome-mediated signalling promotes tumour progression through communication between the tumour and surrounding stromal tissue [6], activation of proliferative and angiogenic pathways [5,7], by bestowing immune suppression [8,9], and initiation of pre-metastatic sites [10]. The factors and stimuli that regulate exosome release are not fully understood, although roles have been reported for p53 [11,12], ceramide synthesis [13], calcium signalling [14], and acidosis [15].

Hypoxia is an important feature of the tumour microenvironment that arises due to an imbalance in the supply and consumption of oxygen by tumour cells [16]. Hypoxic tumours exhibit more aggressive phenotypes and are associated with poor patient outcome in a wide variety of cancers [17]. The cellular response to hypoxia is largely mediated by the hypoxia-inducible factor (HIF) family of transcription factors, and results in global transcriptional changes in gene expression, including genes with roles in promoting tumour progression, angiogenesis and metastasis [16,18]. The HIF family isoforms (HIF-1α, -2α and -3α) are targeted for degradation under normal oxygen conditions (normoxia; 21% O_2_) by the action of specific O_2_-, iron- and 2-oxoglutarate dependent prolyl hydroxylases [16]. Inhibition of these prolyl hydroxylases under normoxic conditions therefore prevents degradation of the HIF family, allowing them to bind and regulate their transcriptional target genes [19].

Recent evidence has highlighted a role for hypoxic tumour cell-derived exosomes in promoting angiogenic signalling [20,21], and there is evidence in several systems for enhanced microvesicle or microparticle release under hypoxia [22,23] and anoxia [24]. Given the observations which independently link both hypoxia and exosome-mediated signalling to invasive tumour phenotypes, it is of interest to investigate if hypoxia might promote tumour progression through altered exosome release. Here we present data which demonstrate that breast cancer cells exposed to hypoxia release higher numbers of exosomes than cells under normoxia, and that this may be mediated by the HIF oxygen sensing system.

## Methods

### Cell culture

The breast cancer cell lines MCF7, SKBR3, and MDA-MB 231 were cultured at 37°C in a 5% CO_2_ humidified environment as adherent monolayers in RPMI 1640 media (Sigma-Aldrich) supplemented with 5% fetal calf serum (FCS) (Sigma-Aldrich), RPMI 1640 with 10% FCS, or DMEM media (Sigma-Aldrich) with 10% FCS respectively. For exosome isolation, cells were cultured in media supplemented with exosome-depleted FCS. FCS was depleted of bovine exosomes by ultracentrifugation at 100,000 × *g* for 16 hours at 4°C. Cell counts were performed using a haemocytometer and viability was determined by 0.1% (w/v) Trypan blue (Sigma-Aldrich) exclusion. Transfections were performed using Lipofectamine 2000 (Invitrogen) as described by the manufacturer. HIF-1α siRNA (sense 5^′^-CUGAUGACCAGCAACUUGAdTdT-3^′^ and antisense 5^′^-UCAAGUUGCUGGUCAUCAGdTdT-3^′^) and negative control siRNA (sense 5^′^-UUCUCCGAACGUGUCACGUTT-3^′^ and antisense 5^′^-ACGUGACACGUUCGGAGAATT-3^′^) were purchased from Shanghai GenePharma and used at a final concentration of 20 nM as previously described [25].

### Hypoxic exposure

Hypoxic experiments were performed within a Hypoxic Glove Box (Coy Laboratory Products) at either 1% or 0.1% O_2_ at 37°C in a 5% CO_2_ humidified environment with the balance provided by nitrogen. Alternatively, cells were treated with the HIF hydroxylase inhibitor dimethyloxalylglycine (DMOG) (Enzo Life Sciences) at a final concentration of 1 mM.

### Exosome isolation

To assess exosome release, cells were seeded at least 24 hours prior to hypoxia or other treatments to allow cells to attach and achieve a growth phase. After culture in the presence or absence of hypoxia, conditioned media was harvested for exosome isolation. Exosomes are traditionally isolated from conditioned media by serial centrifugation at low speed, followed by ultracentrifugation at 100,000 × *g* to pellet the exosomes [26,27]. Recently a proprietary method of exosome isolation called Exoquick^TM^ has been made commercially available, which is reported to provide a rapid and efficient method for exosome isolation [28]. Conditioned media underwent serial centrifugation (300 × *g*; 10 min, 2000 × *g*; 10 min, 10,000 × *g*; 30 min) prior to exosome isolation by ultracentrifugation (100,000 × *g*; 70 min) or Exoquick^TM^ precipitation. Exosome precipitation with the Exoquick^TM^ reagent (System Biosciences) was performed according to the manufacturer’s instructions. Pelleted exosomes were resuspended in phosphate buffered saline and stored at −20°C. Cell counts and viability were also determined at the time of harvest in order to account for differences in cell growth (Additional File 1).

### Electron microscopy

Exosome fractions were fixed with paraformaldehyde to copper mesh formvar grids (ProSciTech) and immunolabelled with mouse monoclonal anti-human CD63 (BD Pharminigen^TM^) and a gold-labelled (10 nm) goat anti-mouse IgG secondary antibody (Sigma-Aldrich). Grids were further fixed with 1% glutaraldehyde and negatively stained by 0.5% uranyl acetate. Samples were observed using the JEOL 1200EX Transmission Electron Microscope housed at Flinders Medical Centre, Bedford Park.

### Nanosight nanoparticle tracking analysis

Isolated exosomes were analysed using the Nanosight LM10 system (Nanosight Ltd) [29,30] equipped with a blue laser (405 nm). Nanoparticles were illuminated by the laser and their movement under Brownian motion was captured for 60 seconds. For example of video captures, see Additional File 2. Videos were then subjected to nanoparticle tracking analysis (NTA) using the Nanosight particle tracking software to provide nanoparticle concentrations and size distribution profiles. At least three videos were captured for each individual sample to provide a representative concentration measurement, and all analysis settings were kept constant within each experiment. Size distribution profiles obtained from NTA were averaged within each sample across the video replicates, and then averaged across samples to provide representative size distribution profiles. These distribution profiles were then normalised to total nanoparticle concentrations or final cell counts.

### SDS-PAGE and immunoblotting

Exosomal fractions were resolved by polyacrylamide gel electrophoresis and electroblotted onto polyvinylidene difluoride membrane (Millipore). Non-reducing conditions were used for CD63 immunoblots due to the sensitivity of the antibody epitope to reducing conditions, as described previously [26]. Primary antibodies used were mouse monoclonal anti-human CD63 (BD Pharminigen^TM^), mouse monoclonal anti-human CD9 (Santa Cruz Biotechnology), rabbit polyclonal anti-human Tsg101 (Tumour susceptibility gene 101) (Abcam), mouse monoclonal anti-mouse flotillin-1 (BD Transduction Laboratories^TM^), and rabbit polyclonal anti-human cofilin-1 (Cell Signaling Technology). The horseradish peroxidase-conjugated secondary antibodies donkey anti-mouse IgG and goat anti-rabbit IgG (Immunopure) were used with enhanced chemiluminescence detection SuperSignal West Pico (Thermo Scientific). Membranes were visualised with the ImageQuant LAS 4000 system (GE Healthcare Life Sciences) or the BioRad ChemiDoc^TM^ MP Imaging System (Bio-Rad Laboratories). Band intensity quantitation was performed using MultiGauge software (FujiFilm).

### RNA extraction and real-time RT-PCR

Cells and isolated exosomes had RNA extracted using the TRIzol® reagent (Invitrogen). Exosome preparations were spiked with synthetic *Caenorhabditis elegans* miR-54 (*cel* miR-54) prior to homogenisation by TRIzol as previously described [31]. TaqMan miRNA-specific primers and reverse transcription kits (Applied Biosystems) were used to synthesize cDNA. miRNA-specific cDNA was then used for relative quantitation by real-time reverse transcription polymerase chain reaction (real-time RT-PCR) with TaqMan Universal PCR Master Mix and the appropriate TaqMan miRNA assay (Assay IDs: miR-210: 000512, miR-21: 000397, miR-16: 000391, let7a: 000377, RNU6B: 001093, *cel* miR-54: 001361; Applied Biosystems). Each PCR contained 1 μL reverse transcription product and was performed in triplicate using the Corbett Rotor-gene 2000 (Qiagen). Results for cellular RNA reactions were normalised to small nuclear RNA gene RNU6B, while exosomal RNA reactions were normalised to *cel* miR-54 or miR-16. Normalisation and relative expression analysis was performed using the Q-Gene software [32].

### Statistical analysis

Significant differences between normoxic and hypoxic nanoparticle concentrations were determined by the Student T test using the KaleidaGraph software (Synergy Software), with a *P* value of < 0.05 considered to be significant. Error bars represent standard error of the mean (SEM).

## Results

### Exosome isolation by ultracentrifugation and Exoquick^TM^ precipitation

**Figure 1:**
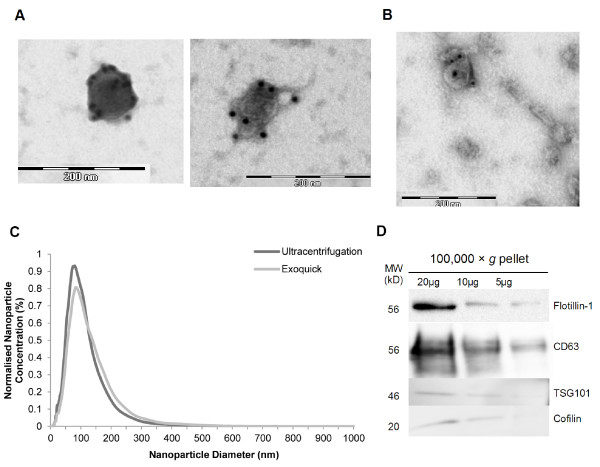
**Exosome isolation by ultracentrifugation and Exoquick.**^**TM**^**precipitation from MCF7 conditioned media.** (**A**, **B**) Transmission electron microscopy and CD63 immunolabelling of MCF7 exosomes isolated by ultracentrifugation (**A**) and Exoquick^TM^ precipitation (**B**). (**C**) Nanoparticle tracking analysis (NTA) of MCF7 exosomes isolated by ultracentrifugation and Exoquick^TM^ precipitation. Data represent the average size distribution profile of n = 4 for each purification method normalised to the total nanoparticle concentrations. Data for each individual sample derive from four different videos and analyses. (**D**) Immunoblotting of ultracentrifugation pellet for exosomal proteins CD63, TSG101, cofilin and flotillin-1.

Exosomes were isolated from the conditioned media of a breast cancer cell line (MCF7) by serial low speed centrifugation followed by either ultracentrifugation or precipitation with the Exoquick^TM^ reagent. To confirm exosomal purification, samples isolated by both methods were examined by transmission electron microscopy and Nanosight NTA (Figure 1). Electron microscopy revealed the presence of vesicles within the expected size range of exosomes (30 – 100 nm) which were positively immunolabelled with CD63-specific gold particle-conjugated antibodies (Figure 1A and 1B), confirming CD63 as an exosomal marker in this system. Exosomes prepared by both methods exhibited similar morphologies and sizes, although Exoquick^TM^ exosomes tended to form long strings of vesicles (Figure 1B), likely as a result of the polymer reagent. NTA of the exosome fractions using the Nanosight microscope revealed the presence of nanoparticles with a modal size of 80 nm and 84 nm for the ultracentrifugation and Exoquick^TM^ samples respectively (Figure 1C), similar to previous reports using this technology [33-36]. Immunoblotting of exosome fractions confirmed the presence of the exosomal proteins CD63, TSG101, flotillin-1 and cofilin (Figure 1D), as identified in the ExoCarta database [37].

### Exoquick^TM^ is an efficient method for exosome isolation from conditioned media

**Figure 2:**
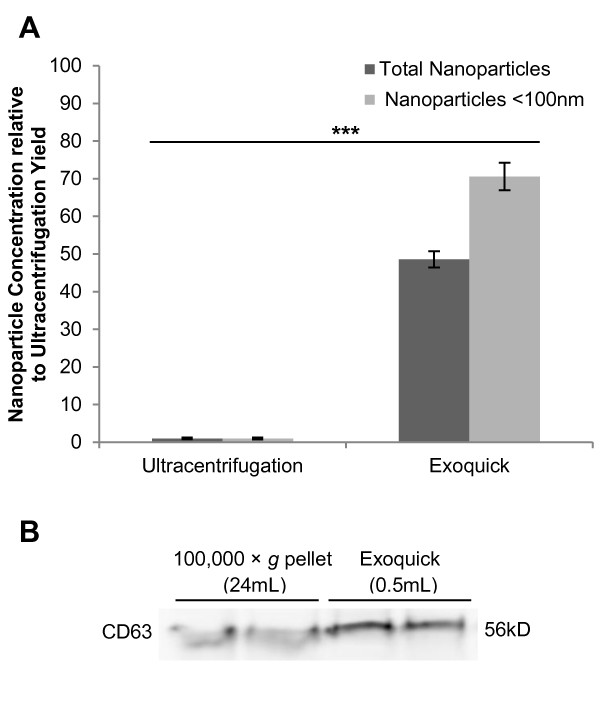
**Comparison of exosome isolation efficiency by ultracentrifugation and Exoquick.**^**TM**^**precipitation.** (**A**) Exosome fractions from ultracentrifugation or Exoquick^TM^ precipitation were analysed by NTA. Nanoparticle concentrations per mL of original volume of media conditioned by 48 hour MCF7 culture (10.5 mL and 0.2 mL for ultracentrifugation and Exoquick^TM^ respectively) were determined, and are expressed here relative to the ultracentrifugation results (n = 8; ± SEM). (**B**) CD63 immunoblot, performed under non-reducing conditions, of exosomes isolated by ultracentrifugation or Exoquick^TM^ precipitation from 24 mL or 0.5 mL MCF7 conditioned media respectively. Exosome pellets were resuspended in equal volumes of PBS for both methods, and loading was controlled by volume. *** corresponds with *P* value < 0.001.

The efficiency of a novel proprietary reagent called Exoquick^TM^ for exosome isolation from small volumes in cell culture systems (< 1 mL) was compared to the traditional method of ultracentrifugation. Nanosight NTA quantitation identified that nanoparticle concentrations were approximately 50-fold higher in exosome fractions isolated by Exoquick^TM^ as compared to ultracentrifugation of the same conditioned media (Figure 2A; *P* < 0.0001). Exoquick^TM^ precipitation yielded a mean concentration of 2.56 × 10^11^ ± 1.13 × 10^10^ nanoparticles per mL of conditioned media, while ultracentrifugation yielded only 5.27 × 10^9^ ± 1.31 × 10^9^ nanoparticles per mL. Nanosight quantitation was supported by immunoblotting for the exosome marker CD63, which demonstrated similar band intensities for ultracentrifugation- and Exoquick^TM^-derived exosome fractions generated from 24 mL and 0.5 mL conditioned media respectively (Figure 2B).

### Hypoxic exposure of breast cancer cells increases exosome concentration in conditioned media

**Figure 3:**
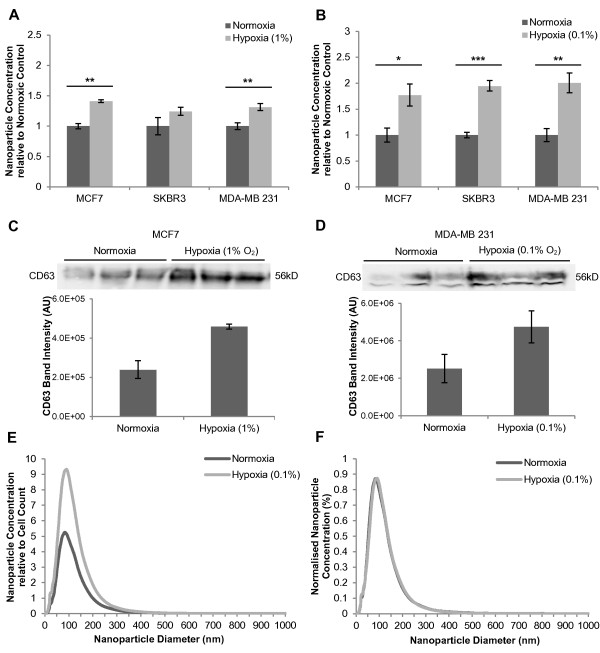
**Hypoxic enhancement of exosome release by breast cancer cells.** (**A**) MCF7, SKBR3 and MDA-MB 231 cells were cultured at 1% O_2_ for 48 hours. Exosomes were isolated from conditioned media by Exoquick^TM^ precipitation. Nanoparticle concentrations were determined by NTA and expressed relative to the normoxic control. Data for each sample were derived from four different videos and analyses (n ≥ 3; ± SEM). (**B**) MCF7, SKBR3 and MDA-MB 231 cells were cultured at 0.1% O_2_ for 24 hours and exosomes were isolated and analysed as described in (A). (**C**) CD63 immunoblot of MCF7 Exoquick^TM^ precipitants from a 48 hour culture under normoxia or 1% O_2_, including band intensity quantitation.(**D**) CD63 immunoblot of MDA-MB 231 Exoquick^TM^ precipitants from a 24 hour culture under normoxia or 0.1% O_2_, including band intensity quantitation_._ (E, F) Nanoparticle size distribution profiles obtained by NTA for hypoxic (0.1% O_2_) MCF7 Exoquick^TM^ precipitants were normalised to final cell counts for normoxia and hypoxia (**E**) and relative nanoparticle size distribution profiles were obtained by normalising to total nanoparticle concentration (**F**). All CD63 immunoblots were performed under non-reducing conditions as described previously [26]. Annotations *, **, and *** correspond with *P* values <0.05, <0.01 and <0.001 respectively.

To examine the impact of hypoxia on exosome release, MCF7 breast cancer cells were exposed to moderate hypoxia (1% O_2_) in a hypoxic glovebox (Coy Laboratories). Exosomes were isolated from the conditioned media after 48 hours and quantitated by Nanosight NTA. Exosome fractions harvested from hypoxic cells demonstrated significantly higher nanoparticle concentrations when compared with exosome fractions from the normoxic control (1.41-fold; *P* = 0.0011) (Figure 3A). To examine whether this enhancement occurred in other breast cancer cell lines, MDA-MB 231 and SKBR3 cells were also studied. Exosome fractions isolated from MDA-MB 231 conditioned media yielded greater nanoparticle concentrations after 48 hour culture at 1% O_2_ (1.32-fold; *P* = 0.0060) (Figure 3A). SKBR3 cells also demonstrated an increased nanoparticle concentration in hypoxic exosome fractions (1.24-fold) (Figure 3A), though this did not achieve statistical significance (*P* = 0.16). To determine if the observed increases in nanoparticle concentration corresponded to exosome levels, Exoquick^TM^ precipitants were assessed for levels of the exosome marker CD63 by immunoblotting (Figure 3C). CD63 was present in protein extracts from exosomes purified in this way and increased CD63 levels were observed in hypoxic exosome fractions after exposure to 1% O_2_ for 48 hours, as shown here for MCF7 (Figure 3C). Band intensity quantitation revealed a significant increase in CD63 band intensity for hypoxic MCF7 exosome fractions (*P* = 0.0096), supporting the observations made using the Nanosight. This increase in CD63 is likely to represent hypoxic enhancement of exosome release as cellular CD63 levels were not enhanced by hypoxic exposure (results not shown). Similar results were observed for immunoblots of SKBR3 exosome fractions for the exosome markers CD63 and CD9, and band intensity quantitation of these immunoblots demonstrated increased CD63 and CD9 in hypoxic exosome fractions (*P* = 0.062 and *P* = 0.017) (Additional File 3A).

To assess whether a more severe hypoxic exposure would affect exosome release in a similar manner, cells were cultured at 0.1% O_2_ for 24 hours, prior to Exoquick^TM^ precipitation and NTA (Figure 3B). Due to the severity of the hypoxic exposure, the duration of exposure was decreased to 24 hours to limit the impact on cell viability and growth (Additional File 1). Nanoparticle concentrations were normalised to cell numbers at the time of harvest where the severe hypoxic exposure resulted in a significant reduction in cell growth, such as for MCF7 and SKBR3 cell line experiments. Exosome fractions isolated from hypoxic breast cancer cells contained significantly higher nanoparticle concentrations per cell count compared to the normoxic control exosome fractions for all three cell lines (Figure 3B). MCF7 culture-derived Exoquick^TM^ precipitants demonstrated a 1.77-fold increase in total nanoparticle concentration (*P* = 0.016) after 24 hours at 0.1% O_2_ (Figure 3B). SKBR3 cells experienced a 1.94-fold increase in total nanoparticle concentration (*P* = 0.00019) and MDA-MB 231 cells released 2-fold more nanoparticles (*P* = 0.0098) (Figure 3B). These data were supported by CD63 immunoblotting of exosomes purified from other breast cancer cell lines under normoxic and hypoxic conditions, MDA-MB 231 (Figure 3D) and SKBR3 (Additional File 3B) which revealed increased levels of CD63 and CD9 present in the hypoxic exosome fractions.

The increased nanoparticle concentrations for the hypoxic exosome fractions corresponded with peaks approximately 80 to 90 nm (Figure 3E), within the expected size range of exosomes. There were no qualitative differences identified between the hypoxic and normoxic exosome size distribution profiles when normalised to total nanoparticle concentrations (for example of MCF7 exosomes see Figure 3F). This suggests that exposure to hypoxia did not affect the size profile of the exosome fractions, and establishes that the observed hypoxic increases in nanoparticle concentrations represent a change in concentration of a similar population of nanovesicles (i.e. exosomes) to that present in the normoxic control.

**Figure 4:**
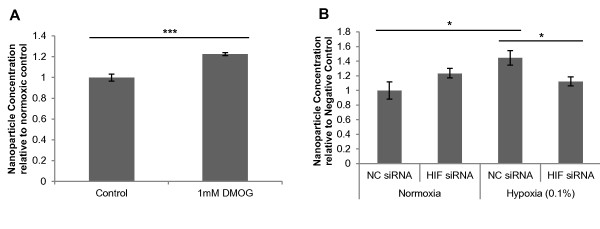
**Exosome release is modulated by induction of hypoxia inducible factor.** (**A**) MDA-MB 231 breast cancer cells were treated with 1 mM DMOG for 24 hours. Exosomes were isolated and quantitated by NTA as previously described (n = 4; ± SEM). (**B**) MDA-MB 231 cells were transfected with negative control (NC) siRNA or siRNA targeting HIF-1α prior to exposure to 0.1% O_2_ for 24 hours. Exosomes were isolated by Exoquick^TM^ and quantitated by NTA (n = 4; ± SEM). Annotations *, **, and *** correspond with *P* values <0.05, <0.01 and <0.001 respectively.

To examine for a potential role of the HIF oxygen sensing system in promoting exosome release, the influence of the HIF hydroxylase inhibitor, DMOG, a 2-oxoglutarate analogue which induces a HIF response, was considered. Treatment of MDA MB-231 cells with 1 mM DMOG for 24 hours resulted in a modest yet significant increase in exosome release as determined by NTA quantitation (1.23-fold; *P* = 0.00095) (Figure 4A). Furthermore, MDA-MB 231 cells transfected with a siRNA targeting HIF-1α failed to show significant enhancement of exosome release after hypoxic exposure (1.12-fold; *P* = 0.39) compared to cells transfected with a negative control siRNA (1.45-fold; *P* = 0.027) (Figure 4B). There was a significant difference in nanoparticle concentration between the HIF siRNA transfection and the negative control siRNA under hypoxia (*P* = 0.031). These data suggest a putative role for HIF signalling in the hypoxic enhancement of exosome release.

### Hypoxic regulation of exosomal miR-210

**Figure 5:**
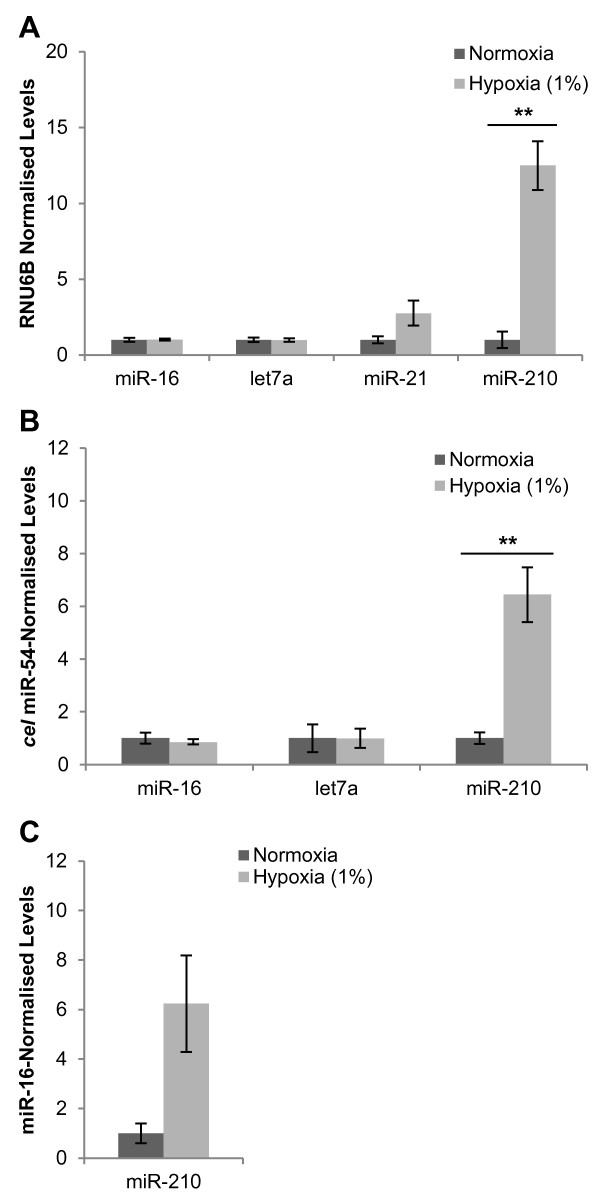
**Cellular and exosomal microRNA expression levels in response to hypoxia.** (**A**) Mean normalised expression levels of MCF7 cellular miRNAs as determined by miRNA-specific Taqman real-time RT-PCR assays in response to 1% O_2_ for 48 hours and normalised to RNU6B. Expression values are presented relative to normoxic control. (n = 3; ± SEM). (**B**, **C**) Exoquick^TM^ precipitants isolated from MCF7 cell culture after 48 hours at 1% O_2_ were spiked with *cel* miR-54 and used for RNA extractions. This exosomal RNA was then assayed for miR-16, let7a, miR-210 and *cel* miR-54 by real-time RT-PCR and normalised to exogenous *cel* miR-54 (B) or endogenous miR-16 (C) (± SEM). Normoxia *n* = 4; Hypoxia *n* = 5. ** corresponds with *P* value < 0.01.

In order to study qualitative differences in exosomes released under hypoxic conditions, several candidate miRNAs were first considered for their suitability as extracellular control genes during hypoxia. MCF7 cellular RNA after moderate hypoxia (1% O_2_; 48 hours) was assayed for the miRNAs miR-16, let7a and miR-21 using miRNA-specific Taqman real-time RT-PCR assays (Figure 5A). These miRNAs were chosen as potential candidates given their previous use as extracellular control genes or reported presence in exosomes [38-41]. The hypoxically regulated miR-210 was also assayed. Mean normalised expression levels of cellular miR-16 and let7a did not change significantly with exposure to hypoxia (1.03-fold (*P* = 0.86) and 0.99-fold (*P* = 0.96) respectively). Expression levels of cellular miR-21 were increased after hypoxic culture (2.77-fold), however this did not achieve statistical significance (*P* = 0.11). Levels of cellular miR-210 demonstrated a significant increase after hypoxic exposure (12.49-fold; *P* = 0. 0025) as previously reported [42].

To determine if miR-210 levels were also elevated in exosomal RNA and to further investigate miR-16 and let7a as extracellular control genes, Exoquick^TM^ precipitants from MCF7 conditioned media after 48 hours at normoxia or 1% O_2_ were spiked with synthetic *Caenorhabditis elegans* miR-54 (*cel* miR-54) to act as an exogenous control [31,41]. RNA isolated from Exoquick^TM^ precipitants were then assayed for miR-210, miR-16, let7a and *cel* miR-54 (Figure 5B). Although miR-16 and miR-210 were amplified successfully from the exosome fractions, let7A failed to amplify, in spite of previous reports of its presence in exosomes [43,44]. When normalised to exogenous *cel* miR-54, exosomal miR-16 levels were relatively consistent between normoxic and hypoxic samples (0.86-fold; *P* = 0.54), and miR-210 levels were significantly higher for hypoxic exosomal RNA samples (6.44-fold; *P* = 0.0026). When miR-210 was normalised to miR-16, the extent of hypoxic induction (6.23-fold) was equally apparent, although this difference was less significant (*P* = 0.052) (Figure 5C).

## Discussion

Hypoxia is an important feature of tumours, and is associated with aggressive tumour phenotypes and poor patient outcomes [17]. Tumours can communicate with surrounding tissue to promote tumour progression and invasion through the release of exosomes [5,6,45,46]. In this study, we investigated the impact of hypoxia, a clinically important feature of tumour progression, on exosome release by breast cancer cells. Here we present evidence that hypoxia enhances the release of exosomes by three different breast cancer cell lines, and that this process may be mediated, at least in part, by the HIF oxygen sensing pathway.

Exosomes were isolated by both ultracentrifugation and Exoquick^TM^ and were identifiable by their morphology and CD63 immunolabelling. These two purification methods were found to be comparable with regard to nanovesicle size and morphology as determined by NTA and electron microscopy. To our knowledge, this is the first such qualitative comparison of cell culture-derived exosomes currently available for these two methods of exosome isolation, although similar observations have been made in a clinical setting [47]. Exoquick^TM^ precipitation was found to be significantly more efficient at isolating exosomes, as determined by direct quantitation by Nanosight NTA, supporting previous comparisons using protein quantitation and immunoblotting [28]. From our experience, the efficiency of Exoquick^TM^ can allow exosome isolation and detection from as little as 10 μL of conditioned media (data not shown).

There is an increasing body of evidence that tumour cell-derived exosomes play important roles in angiogenesis [5], cancer cell invasion [45], metastasis [10] and immunosuppression [8,9] to promote tumour progression. Therefore, understanding the stimuli which promote exosome release by tumour cells is important in understanding tumour development. Here we present evidence that hypoxia promotes the release of exosome-sized nanoparticles. Exposure of breast cancer cells to modest (1%) and severe (0.1%) hypoxia resulted in mean increases of 32.3 ± 4.8% and 90.9 ± 7.1% of exosome-sized nanoparticles harvested from the conditioned media respectively. This is the first report to provide direct exosome quantitation after hypoxic exposure of cells. Previous studies have noted observations of the hypoxic enhancement of secretions of specific proteins, of putative exosomal origin [21,48,49]. Our study offers the possible explanation that the increased concentrations of these proteins may be due to increased release of exosomes under hypoxia. Recent data failed to identify a significant difference in the concentration of exosomes released by hypoxic endothelial cells [36]. This could be explained by the modest hypoxic exposure (2% O_2_) performed, which is consistent with our observation that hypoxic enhancement of exosome release was relative to the severity of the hypoxic treatment. Alternatively, the substantial phenotypic differences between endothelial cells and epithelial tumour cells could also explain a lack of hypoxic enhancement.

HIF induction may play a role in the hypoxic enhancement of exosome release, which was supported here by manipulation of the HIF oxygen sensing pathway using DMOG and siRNA interference. Further circumstantial evidence for this putative role of HIF is provided by HIF-1α dependent secretion of HSP90α by dermal fibroblasts [50], potentially via exosomes [51]. However, it is important to recognise that the modest enhancement of exosome release experienced during HIF activation under normoxia (i.e. DMOG treatment) and incomplete abrogation of hypoxic enhancement by HIF siRNA suggests that other hypoxic responses may be involved.

Given the role for exosomes in tumour progression, increased release of exosomes by hypoxic tumour cells could translate to increased tumour invasion and progression during hypoxia. In addition to the effect of increased exosome numbers, hypoxic tumour-derived exosomes contain various pro-angiogenic factors which allow them to promote angiogenesis and endothelial cell activation [20,21]. However, these studies did not identify if these proteins were an inherent component of exosomal cargo, or were induced by hypoxic exposure. Recent comparison of normoxic and hypoxic endothelial cell-derived exosomes identified that both protein and mRNA exosomal cargo are affected by hypoxia [36].

How exosomal miRNAs might mediate hypoxic signalling requires further investigation. Here we have presented data which suggest that the miRNA miR-210 is elevated in hypoxic exosomes. This could play a role in promoting tumour progression in response to hypoxia, as miR-210 can promote endothelial cell tubulogenesis [52], as well as repressing DNA repair pathways [53]. One interesting possibility is that exosomal miRNAs may promote hypoxic signalling, for example miR-424 or miR-31 activation of HIF-1α independent of hypoxia [54,55]. Of note, miR-424 is induced by hypoxia [54], and has been identified in tumour-derived exosomes [38]. *In vivo* treatment with melanoma-derived exosomes promotes HIF-1α mRNA expression in sentinel lymph nodes [10], highlighting the importance of further studies into exosome-mediated hypoxic signalling.

## Conclusions

Breast cancer cells release greater levels of exosomes when exposed to hypoxia, and this has important implications for how tumour cells might signal to surrounding tissue in the tumour microenvironment. Hypoxic exosomes contained higher levels of miR-210, highlighting the potential for qualitative differences between normoxic and hypoxic exosomes. Given the impact of hypoxia on invasive cellular phenotypes, it will also be important to identify if in addition to secreting higher levels of exosomes, hypoxia promotes tumour growth by increasing the invasive signals from tumour-derived exosomes.

## Abbreviations

*cel* miR-54: *Caenorhabditis elegans* miR-54; DMOG: Dimethyloxalylglycine; FCS: Fetal calf serum; HIF: Hypoxia-inducible factor; miRNA: microRNA; NTA: Nanoparticle tracking analysis; Real-time RT-PCR: Real-time reverse transcription polymerase chain reaction; SEM: Standard error of the mean.

## Competing interests

The authors declare that they have no competing interests.

## Authors’ contributions

HK contributed to study design, data interpretation, carried out the experiments and prepared the manuscript. MM contributed to study design, data interpretation and manuscript revision. JG coordinated the study design, data interpretation and manuscript revision. All authors have read and approved the final manuscript.

## Pre-publication history

The pre-publication history for this paper can be accessed here:

http://www.biomedcentral.com/1471-2407/12/421/prepub

## Supplementary Material

Additional File 1 **Impact of hypoxia on cell growth and viability.** (A, B) MCF7, SKBR3 and MDA-MB 231 breast cancer cells were cultured for 48 hours under normoxia or 1% O_2_. Cell counts were performed for each well after hypoxic exposure (A) and cell viability was determined by Trypan blue exclusion (B) (n=4; ± SEM). (C, D) MCF7, SKBR3 and MDA-MB 231 breast cancer cells were cultured for 24 hours under normoxia or 0.1% O_2_ and cell counts (C) and viability (D) data were obtained as described above (n=4; ± SEM). ** corresponds with *P* value < 0.01. (PDF 13 kb). Click here for file

Additional File 2 **-isolated exosomes.** Representative video of MCF7 exosomes isolated by Exoquick^TM^ precipitation as visualised by Nanosight LM10 microscope using a 405 nm laser. (MPG 2468 kb). Click here for file

Additional File 3 **Hypoxic enhancement of exosome release as detected by CD63 immunoblot.** (A) CD63 and CD9 immunoblot of SKBR3 Exoquick^TM^ precipitants from a 48 hour culture under normoxia or 1% O_2_, including band intensity quantitation. (B) CD63 immunoblot of SKBR3 Exoquick^TM^ precipitants from a 24 hour culture under normoxia or 0.1% O_2_, including band intensity quantitation. All CD63 immunoblots were performed under non-reducing conditions as described previously [16]. (PDF 29 kb). Click here for file
