# Supplementary figures and images for: Hypoxic enhancement of exosome release by breast cancer cells
Source: BMC Cancer. 2012 Sep 24;12:421. doi: 10.1186/1471-2407-12-421 (PMC3488584; doi:10.1186/1471-2407-12-421)

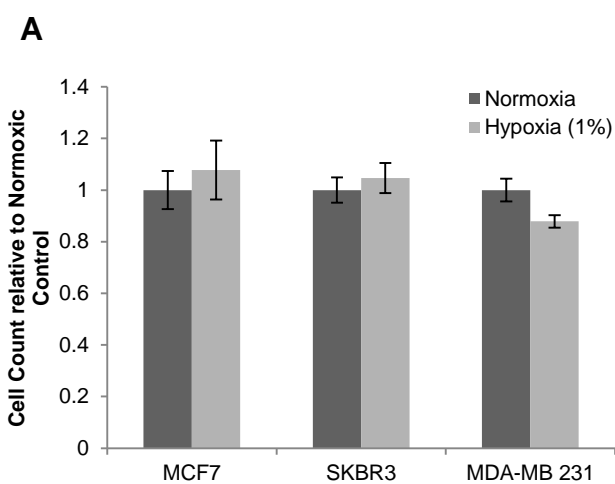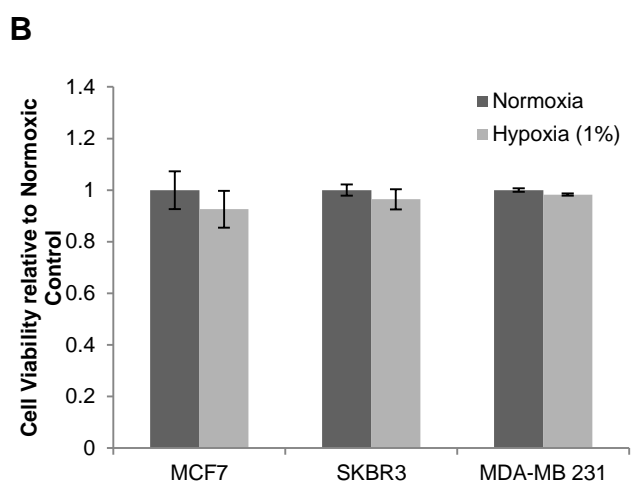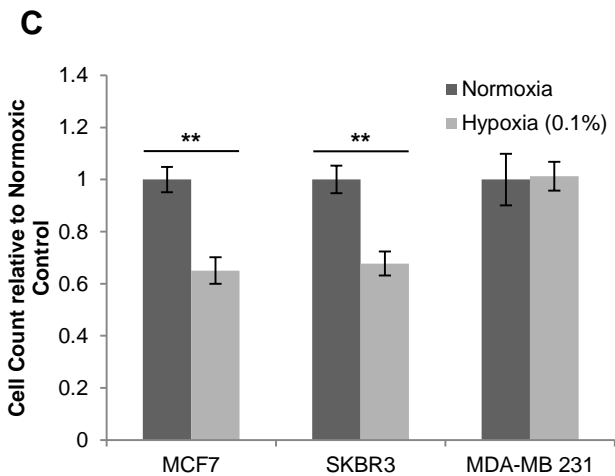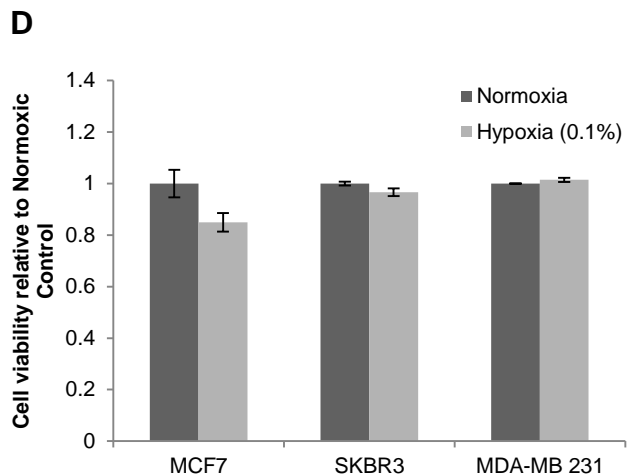

Supplement: Additional File 1 — Impact of hypoxia on cell growth and viability. (A, B) MCF7, SKBR3 and MDA-MB 231 breast cancer cells were cultured for 48 hours under normoxia or 1% O2. Cell counts were performed for each well after hypoxic exposure (A) and cell viability was determined by Trypan blue exclusion (B) (n=4; ± SEM). (C, D) MCF7, SKBR3 and MDA-MB 231 breast cancer cells were cultured for 24 hours under normoxia or 0.1% O2 and cell counts (C) and viability (D) data were obtained as described above (n=4; ± SEM). ** corresponds with P value < 0.01. (PDF 13 kb). [file 1471-2407-12-421-S1.pdf]

**A**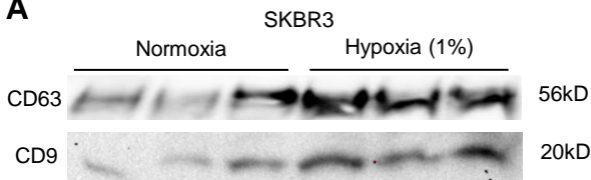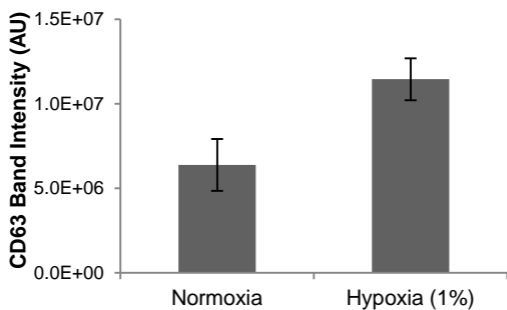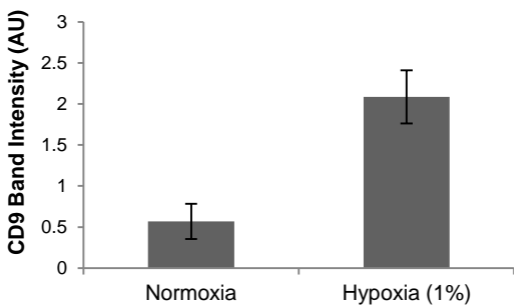**B**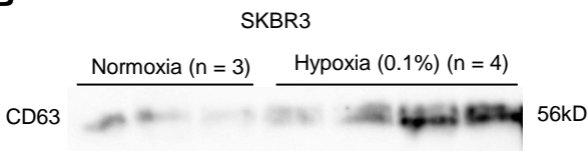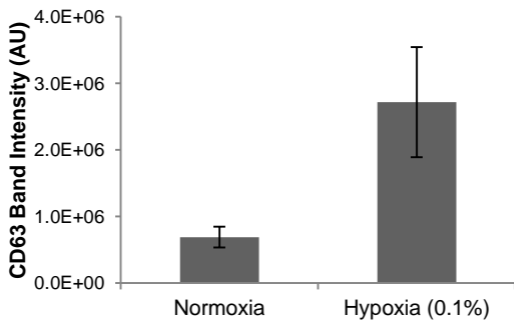

Supplement: Additional File 3 — Hypoxic enhancement of exosome release as detected by CD63 immunoblot. (A) CD63 and CD9 immunoblot of SKBR3 ExoquickTM precipitants from a 48 hour culture under normoxia or 1% O2, including band intensity quantitation. (B) CD63 immunoblot of SKBR3 ExoquickTM precipitants from a 24 hour culture under normoxia or 0.1% O2, including band intensity quantitation. All CD63 immunoblots were performed under non-reducing conditions as described previously [16]. (PDF 29 kb). [file 1471-2407-12-421-S3.pdf]
